# Virus-based vaccine vectors with distinct replication mechanisms differentially infect and activate dendritic cells

**DOI:** 10.1038/s41541-021-00400-w

**Published:** 2021-11-22

**Authors:** Carolina Chiale, Anthony M. Marchese, Yoichi Furuya, Michael D. Robek

**Affiliations:** 1 Department of Immunology and Microbial Disease, Albany Medical College, Albany, NY USA; 2 Present Address: Division of Biological Sciences, University of California San Diego, 9500 Gilman Drive, La Jolla, CA 92093 USA

**Keywords:** Antigen presentation, Viral vectors, Vaccines

## Abstract

The precise mechanism by which many virus-based vectors activate immune responses remains unknown. Dendritic cells (DCs) play key roles in priming T cell responses and controlling virus replication, but their functions in generating protective immunity following vaccination with viral vectors are not always well understood. We hypothesized that highly immunogenic viral vectors with identical cell entry pathways but unique replication mechanisms differentially infect and activate DCs to promote antigen presentation and activation of distinctive antigen-specific T cell responses. To evaluate differences in replication mechanisms, we utilized a rhabdovirus vector (vesicular stomatitis virus; VSV) and an alphavirus-rhabdovirus hybrid vector (virus-like vesicles; VLV), which replicates like an alphavirus but enters the cell via the VSV glycoprotein. We found that while virus replication promotes CD8^+^ T cell activation by VLV, replication is absolutely required for VSV-induced responses. DC subtypes were differentially infected in vitro with VSV and VLV, and displayed differences in activation following infection that were dependent on vector replication but were independent of interferon receptor signaling. Additionally, the ability of the alphavirus-based vector to generate functional CD8^+^ T cells in the absence of replication relied on cDC1 cells. These results highlight the differential activation of DCs following infection with unique viral vectors and indicate potentially discrete roles of DC subtypes in activating the immune response following immunization with vectors that have distinct replication mechanisms.

## Introduction

Vaccination approaches that aim to activate CD8^+^ T cells benefit from using virus-based platforms, as live viral vectors mimic infection with a virus and therefore generate strong cellular immune responses. Virus-based vectors have been widely studied and are currently being tested in clinical trials for vaccine, gene therapy, and oncolytic uses^1–16^. Viral vectors are based on either DNA or RNA viruses, which replicate through different mechanisms and therefore have distinct interactions with the immune system. Despite the extensive evaluation of many viral vectors as preclinical vaccine candidates, it is not always known how these vectors successfully activate a protective immune response.

Specific dendritic cell (DC) subtypes play critical roles in priming immune responses and controlling virus infection, but their functions in generating effective immunity following vaccination with viral vectors are not well understood. Viruses may infect different DC subtypes leading to unique immune activation profiles^17^. Even though direct DC infection can be essential for CD8^+^ T cell priming, antigen cross-presentation mechanisms ensure CD8^+^ T cell activation even in the absence of direct infection^18^. DCs can be subdivided into two subtypes of conventional DCs (cDC1, cDC2), plasmacytoid DCs (pDC), or monocyte-derived DCs (moDC)^19^. cDC1 are associated with strong CD8^+^ T cell activation and are the most efficient subset for cross-presenting antigens^20^, while cDC2 promote the activation of CD4^+^ T cells as well as memory CD8^+^ T cells^21^. pDCs are traditionally known for type I interferon (IFN) production, but these cells also have important antigen presentation functions^22^.

The ability of replicating virus vectors to activate strong immune responses can be attributed to prolonged antigen expression, the induction of cell death that facilitates antigen uptake and cross-presentation by DCs, and their potential to directly infect antigen-presenting cells. When the viruses replicate, they also generate PAMPs and DAMPs that activate different immune receptors, reducing the need for adjuvant. Some viral vectors that are currently studied for vaccination purposes are DNA-based, such as E1-deleted adenovirus and modified vaccinia Ankara, which are immunogenic despite being replication-deficient. We were interested in investigating the way that replicating RNA viruses, such as vesicular stomatitis virus (VSV) and virus-like vesicles (VLV), interact with DCs and prime CD8^+^ T cell responses.

VSV is a negative-strand RNA virus that replicates in the cytoplasm^23^ and encodes a viral protein (M) that interferes with innate immune stimulation^24^. VSV induces strong CD8^+^ T cell responses^25–28^, exists in multiple serotypes, and does not widely infect humans, reducing the possibility of preexisting immunity to the vector^24^. A highly attenuated form of VSV, N4CT1, provides the same immunogenicity as wild-type (WT) VSV but without the potential pathogenicity of a WT virus, and has been tested in clinical trials for Ebola virus and human immunodeficiency virus (HIV)^29–34^. Virus-like vesicles (VLV) are Semliki Forest virus (SFV)-based replicons that propagate in the cytoplasm and produce infectious spherules containing only one structural protein, the VSV-G glycoprotein, which promotes vesicle budding and spread from infected cells^35^. These hybrid vectors were engineered to express foreign antigens that can act as vaccine platforms^36–38^, and like attenuated VSV, VLV vectors are immunogenic but not pathogenic^35,36,39–42^. The VLV platform replicates like an alphavirus; the newly translated SFV RNA replicase synthesizes complementary negative-strand RNA, full-length positive-strand RNA, and a smaller mRNA encoding a foreign antigen and VSV-G^39,43^. Alphavirus replication compartments are formed as small vesicles (spherules) at the plasma membrane where the RNA is synthesized. The replicase complex appears to be on the cytoplasmic side of the spherule neck, and the spherules are then internalized to form large cytopathic vacuoles^36,44^. Overall, the replicase complex is thought to protect the viral nucleic acids from immune receptors in the cytoplasm^44^. In previous studies, we and others showed immunogenicity of VLV and VSV expressing antigens from hepatitis B virus (HBV) and HIV in mice and non-human primates, respectively^37,41,45–48^.

In this work, we investigated the role of viral replication in DC activation and CD8^+^ T cell priming. We took advantage of VLV and VSV entering cells through the same receptor but replicating through different mechanisms to investigate the impact of viral replication on DC activation and CD8^+^ T cell priming. We found that for VSV, viral replication is needed to activate DCs and promote functional CD8^+^ T cell priming. However, VLV activates DCs even in the absence of replication and primes functional CD8^+^ T cells through cDC1.

## Results

### RNA viral vectors differentially infect and activate dendritic cells

**Fig. 1:**
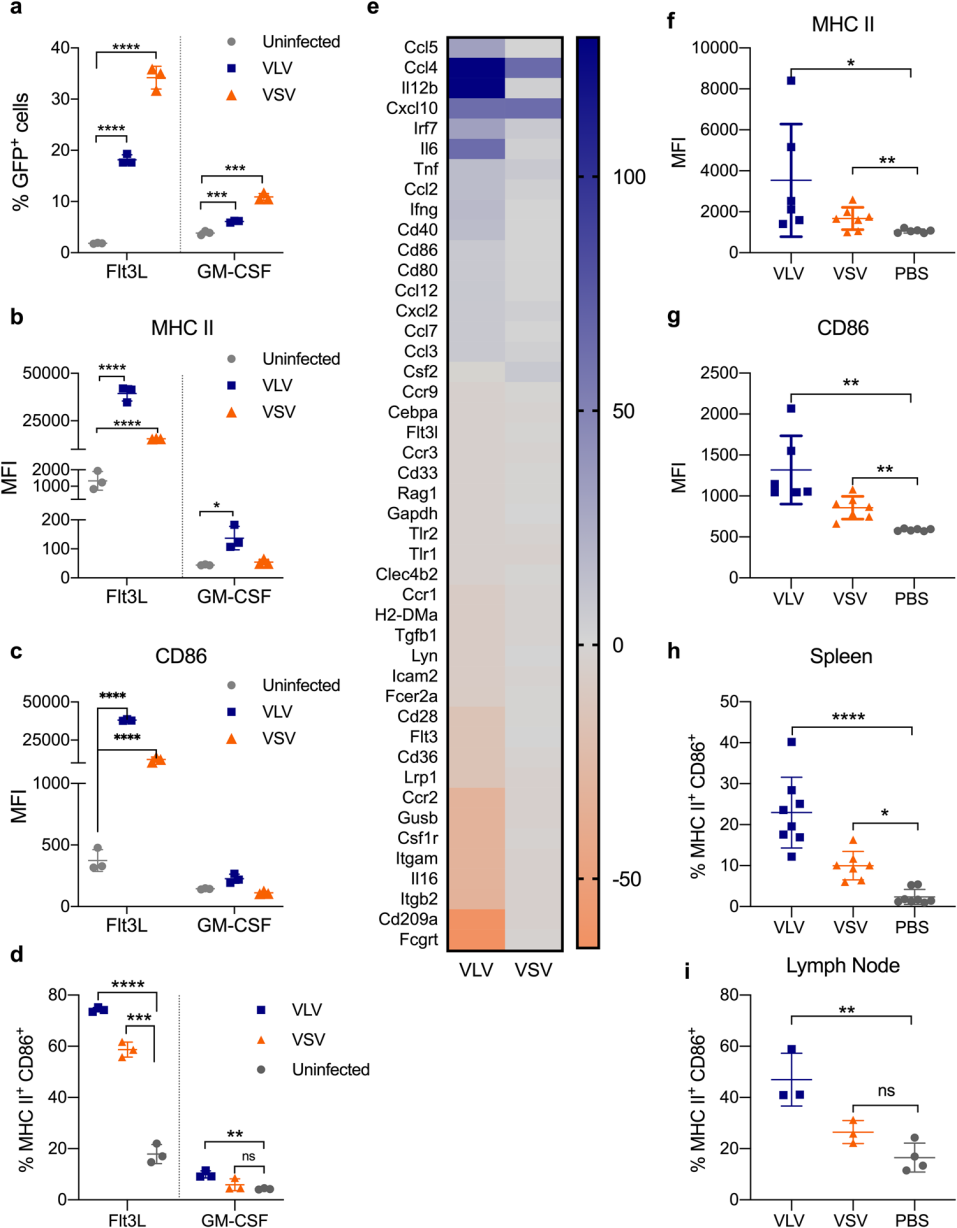
Flt3L and GM-CSF BMDCs become differentially activated following infection with VLV or VSV. **a–d** Flt3L and GM-CSF DCs were infected with VLV or VSV expressing GFP for 18 h (MOI 10). After infection, cells were analyzed by flow cytometry for detection of GFP and activation markers. **a** Percentage of GFP^+^ cells represents the percentage of infected cells. **b** MHC II and **c** CD86 DC activation markers after infection with VSV or VLV. **d** Percentage of MHC II^+^ and CD86^+^ cells. **e** RT^2^ PCR RNA analysis of genes associated with antigen presentation in Flt3L DCs 6 h post-infection relative to uninfected control. **f–i** C57BL/6 mice were infected intramuscularly with VLV or VSV, and 24 h later, spleen (**f–h**) and lymph nodes (**i**) were harvested to analyze cell activation. Cells were selected as CD3^−^, CD19^−^, CD11c^+^ and then analyzed for activation marker expression. **f** MHC II and **g** CD86 DC activation markers in cells from the spleen after infection with VSV or VLV. **h** Percentage of MHCII^+^CD86^+^ cells in the spleen of immunized mice. **i** Percentage of MHCII^+^CD86^+^ cells in pooled lymph nodes from immunized mice. Data are expressed as mean ± standard deviation. *p < 0.05, ***p < 0.001, ****p < 0.0001 compared to uninfected cells or PBS treated mice by multiple *t*-test or one-way ANOVA.

VSV and VLV are RNA-based vectors that replicate in the cytoplasm of infected cells. To investigate the capacity of these vectors to infect and activate antigen-presenting cells (APCs), we generated bone marrow-derived DCs (BMDCs) using either GM-CSF or Flt3L and infected them with vectors expressing GFP. GM-CSF treatment of bone marrow precursors gives rise to a heterogeneous population of APCs in which macrophages are predominant^49^, while Flt3L stimulation causes differentiation into both cDCs and pDCs^50,51^. While both vectors infected DCs, VSV showed higher infection rates compared to VLV (Fig. 1a). Additionally, Flt3L DCs were more susceptible to virus infection than GM-CSF-derived APCs, which is consistent with previous VSV studies^52^. BMDCs were activated after infection with both viral vectors, but VLV caused the strongest increase of activation markers such as MHC II (Fig. 1b and d, Supplementary Fig. 1) and CD86 (Fig. 1c, d). Consistent with lower VLV infection levels, GM-CSF APCs were activated post-VLV infection but to a lesser extent than Flt3L DCs (Fig. 1a–d).

To better understand the impact of virus infection on antigen presentation-related gene regulation, we measured the mRNA expression of genes related to antigen processing and presentation in Flt3L DCs 6 h after infection with VLV or VSV using an RT^2^ profiler PCR assay (Qiagen). This assay measures 84 key genes involved in antigen presentation, and data were normalized to uninfected controls. We focused on Flt3L DCs because of their stronger activation profile. VLV had a broader impact on gene expression in DCs after infection, upregulating genes related to inflammation (such as Ccl5, Ccl4, and IL12b), but also downregulating other genes (Fig. 1e). Specifically, there was a significant increase in pro-inflammatory cytokines such as IL-12, TNF-α, and IL-6, as well as activation markers such as CD40, CD80, and CD86 relative to the uninfected control (Fig. 1e), consistent with the high expression of some markers detected by flow cytometry (Fig. 1c). In contrast, VSV caused only minor gene activation, which is consistent with the expression of the M protein that blocks mRNA nuclear export.

Because the vectors showed strong DC activation in vitro, we next determined the in vivo activation of APCs in mice following infection. We infected mice intramuscularly with VLV, VSV, or PBS, and determined APC activation 24 h post-infection. Consistent with cultured BMDCs, both vectors activated CD11c^+^ APCs, but VLV induced higher activation levels both in spleen cells (Fig. 1f–h, Supplementary Fig. 2) and lymph nodes (Fig. 1i). Together, these data emphasize the differences in DC activation capacity of the two vectors.

**Fig. 2:**
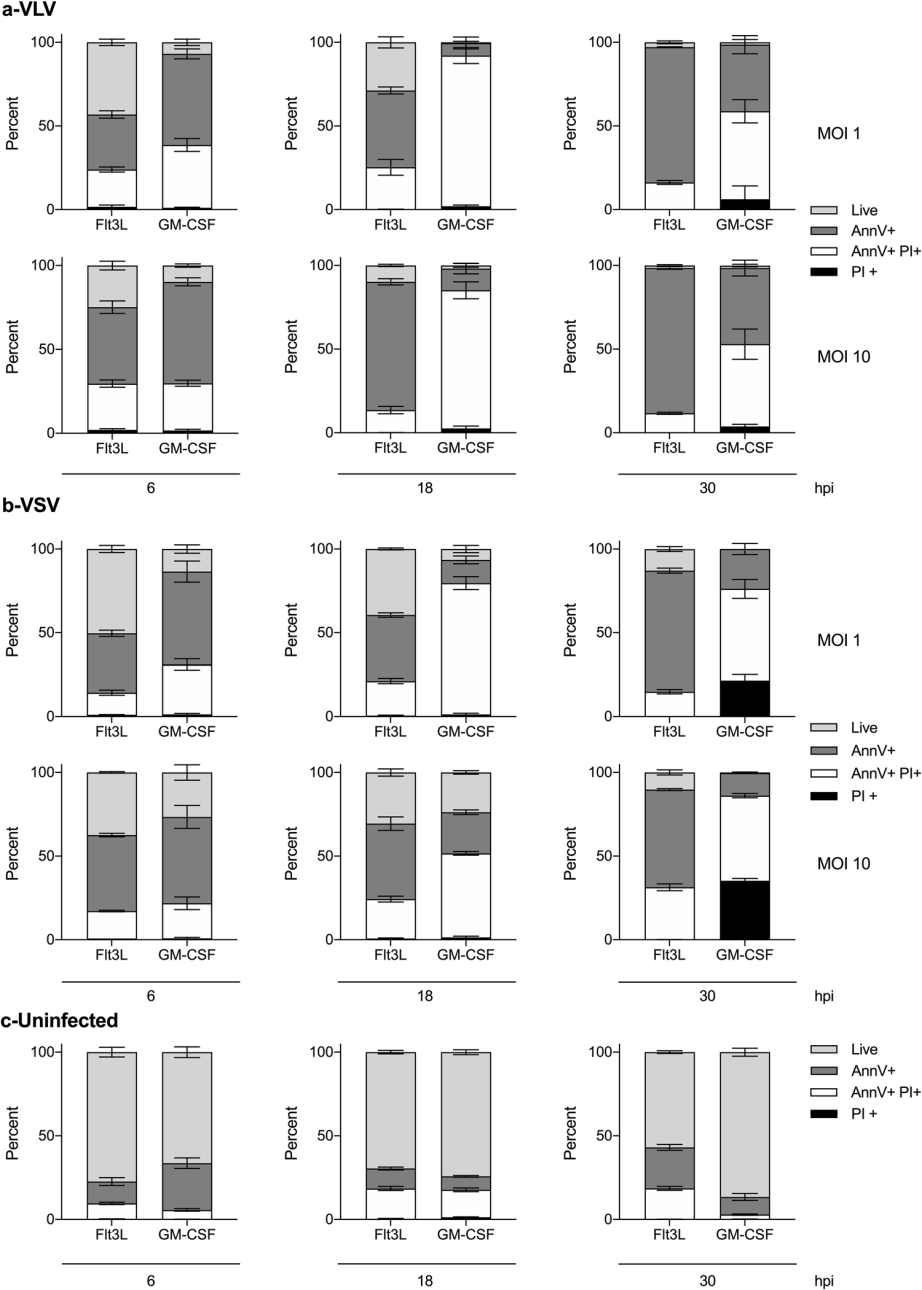
VLV and VSV infection induces similar levels of DC death. Flt3L and GM-CSF DCs were infected with VLV or VSV (MOI 1 or MOI 10) as indicated, and analyzed at 6, 18, and 30 h post-infection. Frequency of cells in early apoptosis (Annexin V+, PI–), late apoptosis (Annexin V+, PI+), dead (Annexin V−, PI+), or live (Annexin V−, PI−) in cells infected with **a** VLV, **b** VSV, or **c** uninfected cells. The graphs represent the average of technical triplicates. Data are expressed as mean ± standard deviation. The VLV and VSV infections were performed concurrently in the same experiments. This figure is representative of two independent experiments.

The lack of gene modulation with VSV vectors could also suggest that VSV is causing rapid cell death, and therefore no cell activation is observed. To evaluate whether the differences in cell activation could be explained by virus-induced cell death, we measured cell death at several times post-infection and multiple MOI. VLV and VSV caused similar levels of cell death over time in Flt3L DCs, and no major differences between the vectors at various MOI were observed (Fig. 2, Supplementary Fig. 3). Interestingly, GM-CSF APCs showed higher susceptibility to virus-induced cell death compared to Flt3L DCs (Fig. 2), and in this cell type, VLV infection resulted in even higher cell death than VSV, which might explain the differences in infection among those cell types and is consistent with previous findings with VSV^52–54^.

**Fig. 3:**
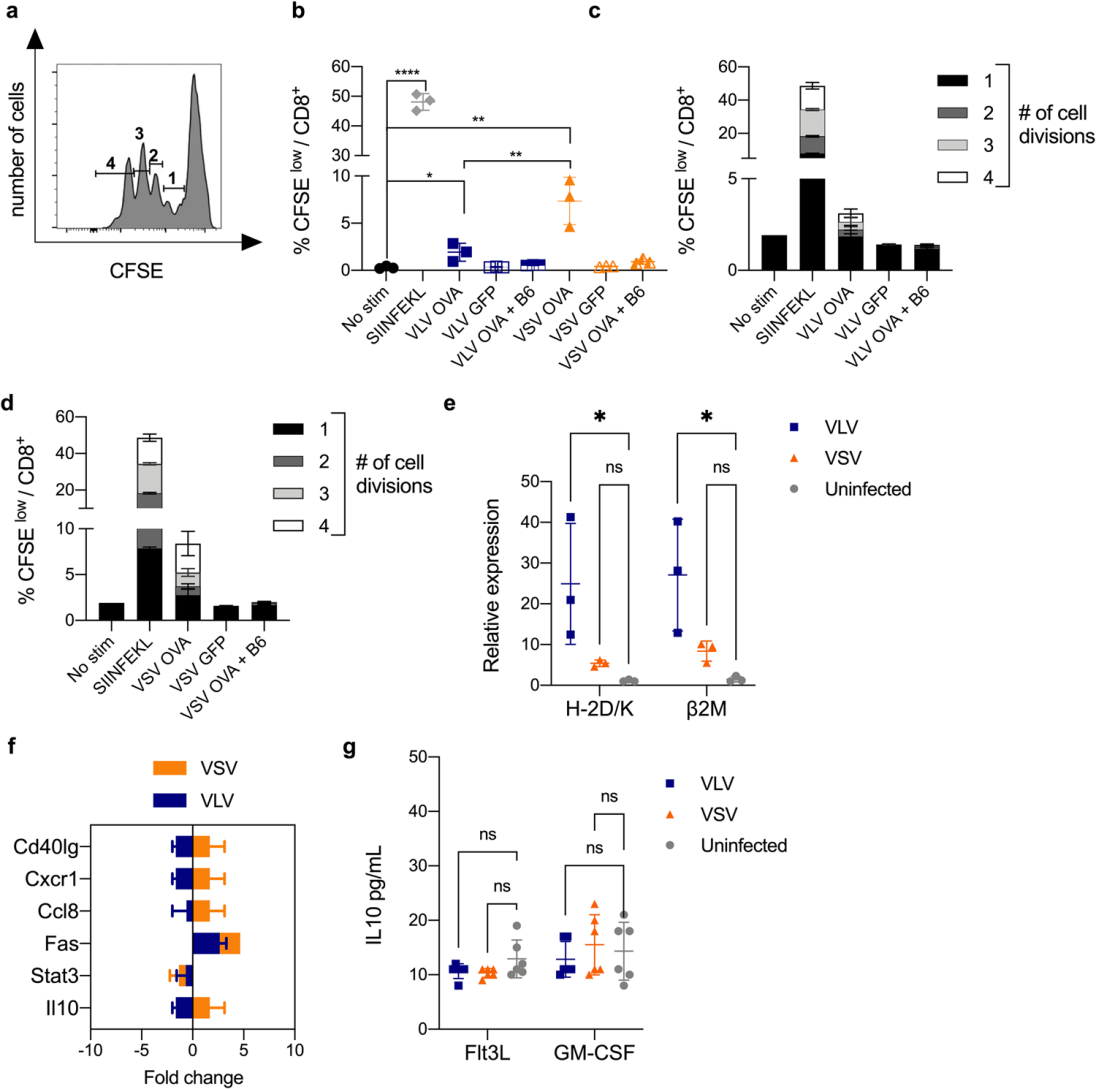
VLV- and VSV-infected BMDCs activate antigen-specific CD8^+^ T cells in vitro. **a** Flt3L-derived BMDCs were infected with VLV or VSV expressing OVA (MOI 10) for 18 h. A positive control consisted of incubating DCs with SIINFEKL peptide for 18 h. After infection or SIINFEKL incubation, cells were washed and co-cultured with CFSE-labeled OT-I splenocytes for 72 h (Ratio = 1:30). **b** Proliferation of SIINFEKL-specific cells was measured by loss of CFSE fluorescence intensity by flow cytometry. Percentage of **b** total proliferation (CFSE^low^ in CD8 + cells) and the number of cell divisions in co-cultures of OT-I cells with **c** VLV or **d** VSV-infected DCs. **e** H-2K/D and B2M RNA levels were measured by RT-qPCR normalized to GAPDH. **f** RT^2^ PCR RNA analysis of IL-10 and other unchanged genes associated with antigen presentation in Flt3L DCs 6 h post-infection relative to uninfected control. **g** IL-10 production by DCs 18 hpi with VLV, VSV, or uninfected control was measured by ELISA. Data are expressed as mean ± standard deviation. *p < 0.05, **p < 0.01, ****p < 0.0001 compared to uninfected control by unpaired *t*-test, one- or two-way ANOVA. The VLV and VSV infections were performed concurrently in the same experiments.

DCs respond to virus infection by increasing expression of activation markers that modulate immune responses. We next examined the capacity of virus-infected cells to prime antigen-specific CD8^+^ T cell responses. We infected DCs with VLV-ovalbumin (OVA) or VSV OVA for 18 h. Cells were then washed and co-cultured with CFSE-labeled OT-I splenocytes for 72 h (Fig. 3a, Supplementary Fig. 4). DCs infected with VLV-OVA stimulated proliferation of OVA-specific CD8^+^ T cells (Fig. 3b, c), but VSV-infected cells promoted greater proliferation of OVA-specific CD8^+^ T cells (Fig. 3b–d), with most cells undergoing four rounds of division. Importantly, the responses were antigen-specific, since neither infection with VLV or VSV expressing GFP nor incubation with non-transgenic splenocytes led to proliferation of OVA-specific CD8^+^ T cells (Fig. 3b–d). It is important to note that we did not separate live from dead DCs for this stimulation, which likely correlates with the low levels of proliferation observed. These results suggest that differences in BMDC infection with VSV or VLV lead to differences in CD8^+^ T cell priming. Surprisingly, VSV promoted stronger CD8^+^ responses than VLV, despite causing lower DC activation compared to VLV. Because MHC I antigen presentation is key for CD8^+^ T cell activation, we investigated the levels of MHC I and B2M expression in infected DCs. Interestingly, VSV-infected cells had lower H-2D/K and B2M mRNA expression than VLV-infected cells (Fig. 3e). We then assessed if the differences in CD8^+^ T cell activation could be due to higher production of inhibitory cytokines such as IL-10 by VLV-infected cells, but there was no significant IL-10 production in any case (Fig. 3f, g). Together, these data suggest higher DC activation with VLV, but greater CD8^+^ T cell priming with VSV, which is not explained by cell activation or inhibitory cytokine production.

### VLV, but not VSV, infection causes bystander DC activation

**Fig. 4:**
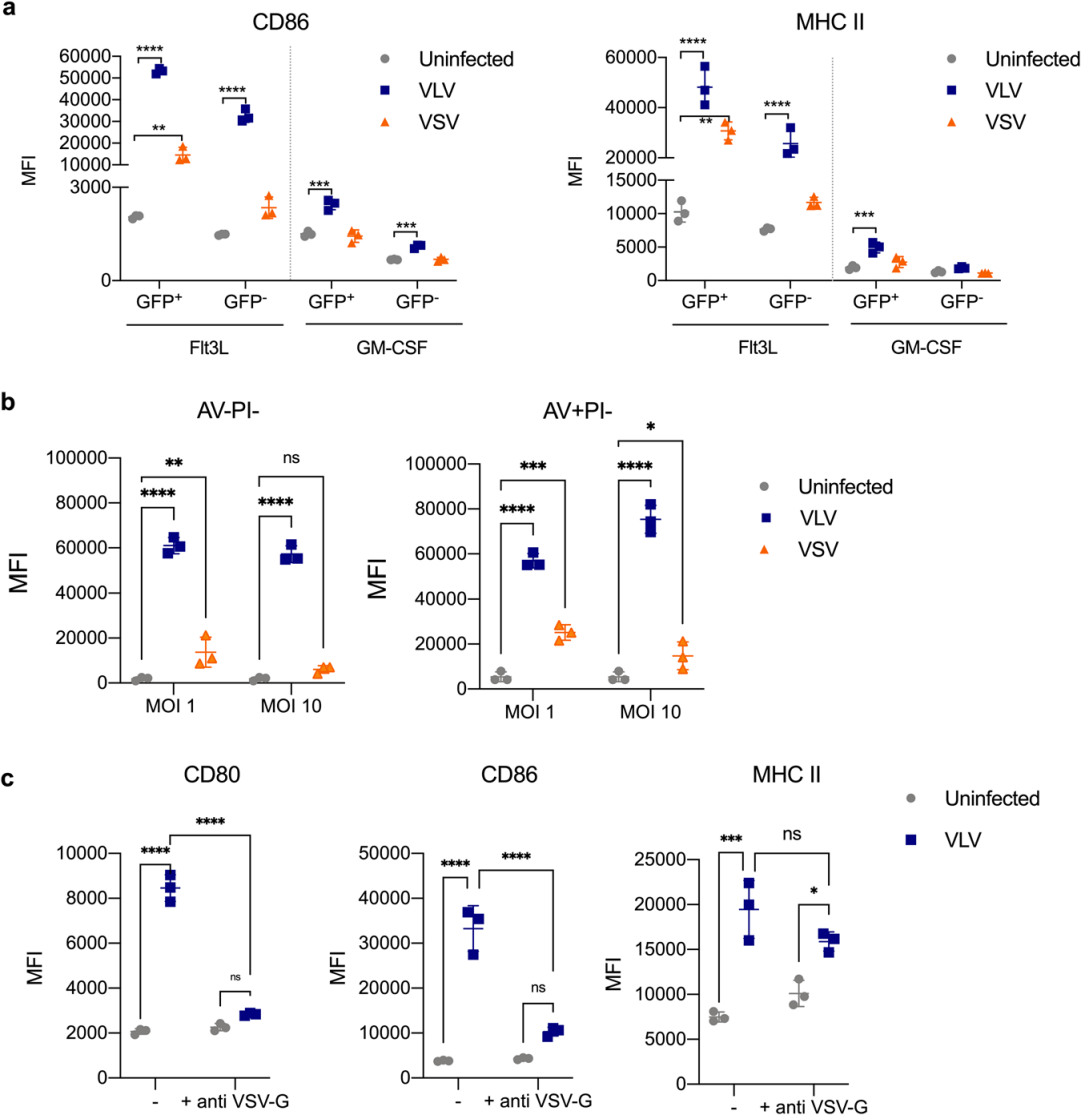
Bystander activation of DCs with VLV but not VSV. Flt3L and GM-CSF DCs were infected with VLV or VSV (MOI 10) for 18 h. After infection, surface expression of the activation markers **a** CD86 and MHC II was analyzed in both infected (GFP^+^) and uninfected (GFP^−^) cells by flow cytometry. **b** Surface expression of CD86 in live cells (AV− PI−) and early apoptotic cells (AV+ PI−). **c** VLV was pre-incubated with anti-VSV antibody prior to infection of Flt3L DCs, and CD80, CD86, and MHC II were measured 18 h post-infection. Data are expressed as mean ± standard deviation. **p < 0.01, ***p < 0.001, ****p < 0.0001 compared to uninfected control by one- or two-way ANOVA.

Infection with both viral vectors led to strong DC activation, particularly with VLV, even though only a fraction of the cells were infected. We reasoned that after infection, bystander activation of uninfected cells could explain the differences in the magnitude of activation. To determine if VLV or VSV induced bystander activation of DCs, we infected cells with vectors expressing GFP. After infection, we stained for activation markers and gated on GFP^+^ (infected) and GFP^−^ (uninfected) cells. Infection with VLV, but not VSV, led to bystander DC activation (Fig. 4a), potentially explaining the stronger DC activation observed with VLV. Also consistent with bystander effects, DC activation was observed both in cells that were alive and in early apoptosis (Fig. 4b). Further, activation was mostly blocked by preincubation of the VLV with antibody to VSV-G (Fig. 4c), suggesting a mechanism whereby DC activation requires initial VSV-G-dependent infection.

### DC activation is independent of IFN signaling

**Fig. 5:**
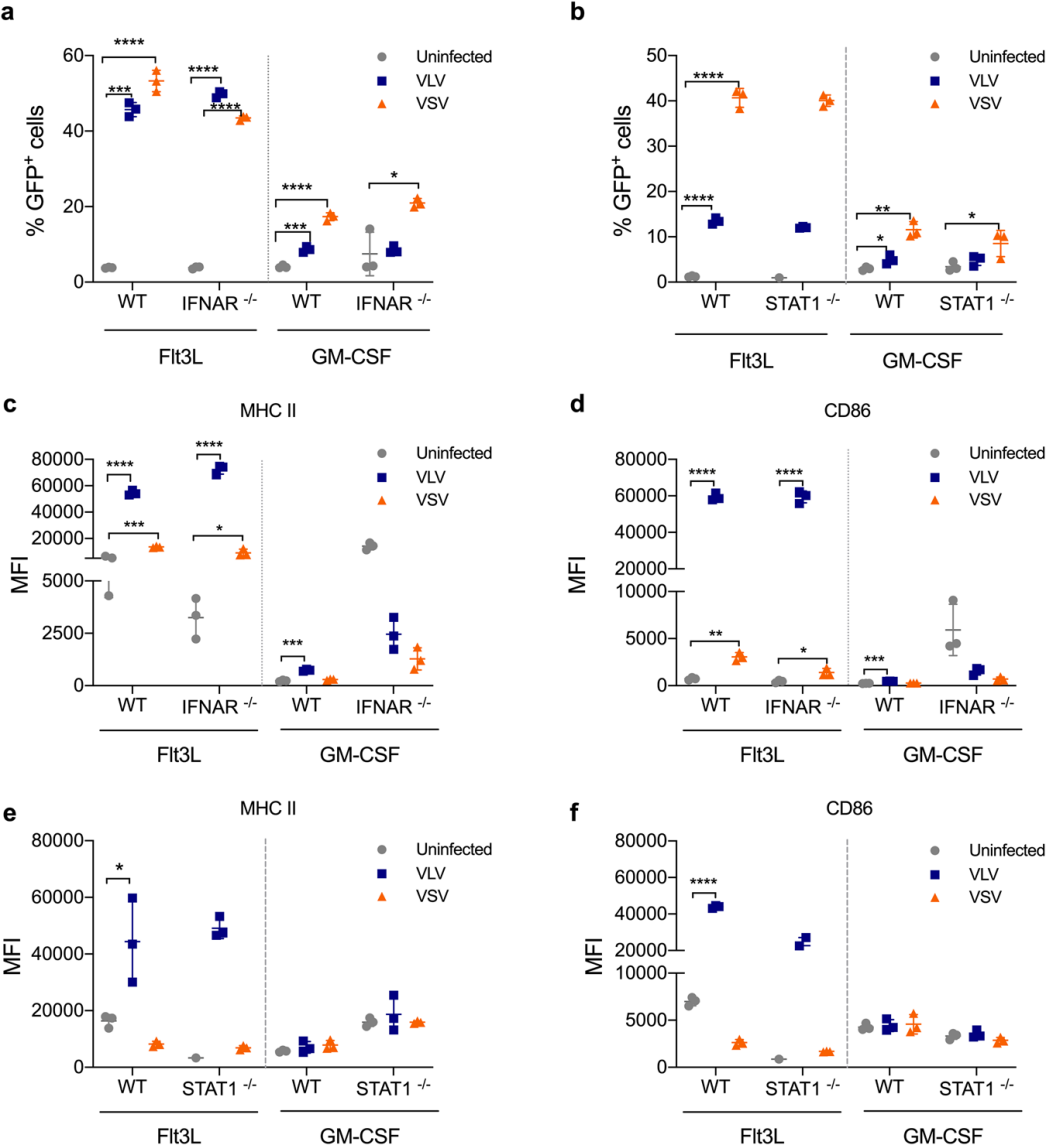
Flt3L DC infection and activation is independent of the type I IFN response. **a**, **b** BMDCs were generated from WT, IFNAR^−/−^, and STAT1^−/−^ mice, and then infected with VLV or VSV for 18 h (MOI 10). GFP expression in Flt3L and GM-CSF BMDCs from **a** IFNAR-deficient or **b** STAT1-deficient mice. **c**, **d** Expression of **c** MHC II and **d** CD86 on IFNAR^−/−^-derived Flt3L BMDCs after VLV or VSV infection. **e**, **f** Expression of **e** MHC II and **f** CD86 activation markers after infection of STAT1^−/−^ Flt3L BMDCs. Data are expressed as mean ± standard deviation. *p < 0.05, **p < 0.01, ***p < 0.001, ****p < 0.0001 compared to uninfected control by one- or two-way ANOVA.

The ability of VLV to induce DC bystander activation led us to determine whether infected cells could secrete a soluble mediator that promoted activation of uninfected cells. Because viral infection can induce expression of antiviral cytokines such as IFN that may influence virus replication or cell activation, we investigated the impact of IFN signaling on DC activation following VSV or VLV infection. We generated BMDCs from IFNAR^−/−^ mice and measured infection and activation. We found that lack of IFNAR did not affect infectivity with VLV or VSV (Fig. 5a), or cell activation (Fig. 5c, d). This result led us to determine whether other interferons could be involved in DC activation by VLV or VSV, and so we utilized cells from mice that lack STAT1 and therefore cannot signal through any interferon. Similar to cells from IFNAR^−/−^ mice, no differences in infectivity or cell activation were observed in STAT1^−/−^ DCs (Fig. 5b, e, f). Together these data suggest DC activation with VLV and VSV is independent of interferon signaling.

### VLV replication is not required for DC activation

**Fig. 6:**
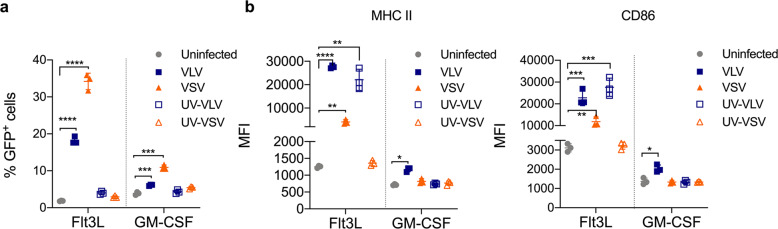
Viral replication is not required for activation of Flt3L-derived BMDCs. Eighteen hours after infection of Flt3L and GM-CSF BMDCs with either VLV, VSV, or UV-inactivated VLV or VSV (MOI 10), cells were analyzed by flow cytometry. **a** Percentage of GFP^+^ cells. **b** MHC II and CD86 expression 18 h after infection of Flt3L- or GM-CSF-derived DCs. Data are expressed as mean ± standard deviation. *p < 0.05, **p < 0.01, ***p< 0.001, ****p < 0.0001 compared to uninfected control by multiple *t*-test or one-way ANOVA.

VLV and VSV both utilize VSV-G to enter cells but have distinct replication mechanisms. We next investigated whether viral replication was important for virus-induced cell activation. To address this, we UV-inactivated the vectors rendering them replication-deficient, and infected cells with either replication-competent or replication-deficient vectors. As the viral vectors do not carry the GFP protein, GFP would only be observed if newly expressed within the infected cells (Fig. 6a). Lack of vector replication was further verified by infection of BHK cells with the same UV-treated vectors, and cells were then observed for up to 3 days post-infection and no signs of infection were detected (GFP expression or morphological changes). Interestingly, VLV replication was not needed for DC activation, while VSV replication was required to activate DCs (Fig. 6b).

### UV-inactivated VLV primes antigen-specific CD8^+^ T cells

**Fig. 7:**
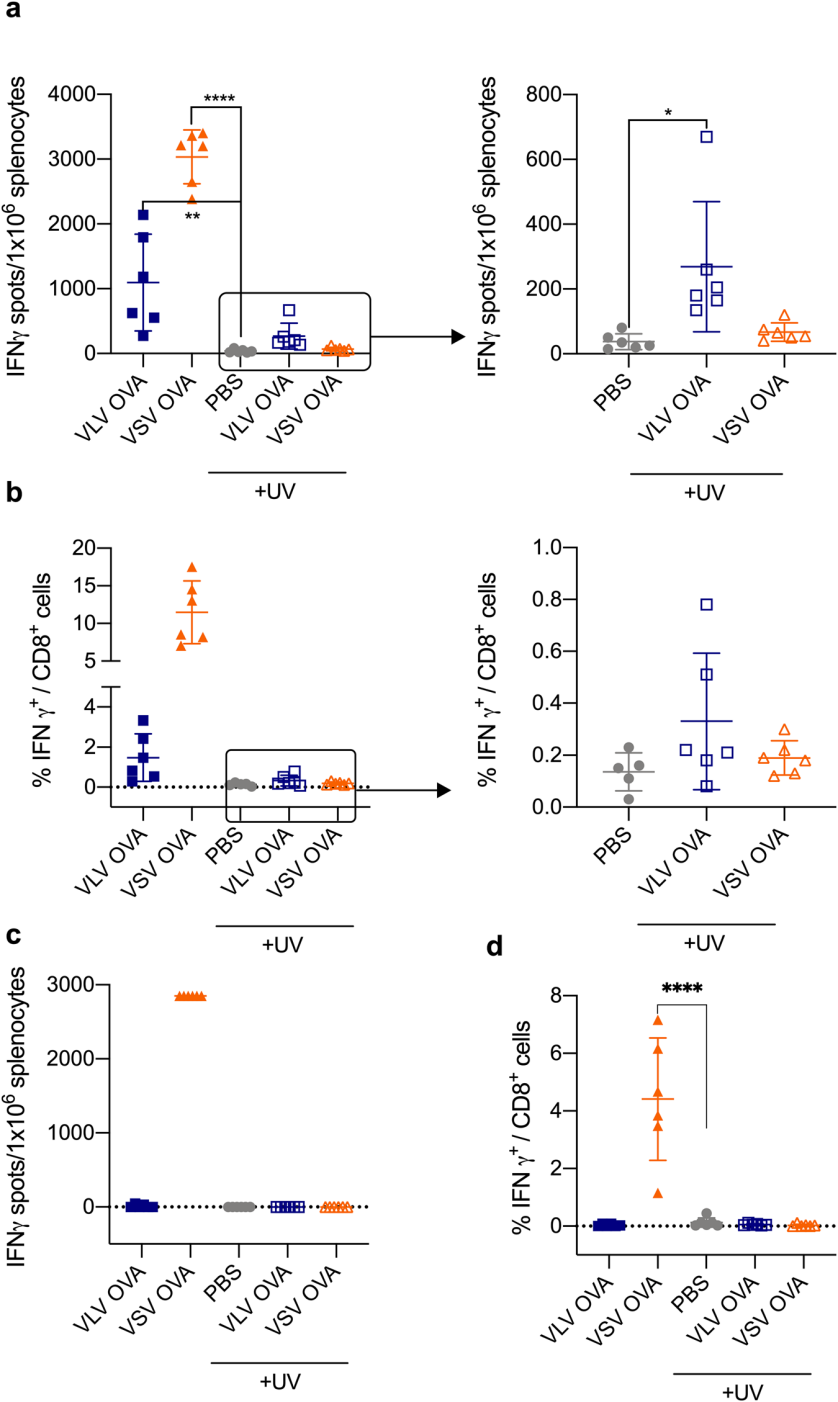
UV-inactivated VLV primes antigen-specific CD8^+^ T cells. Mice received spleen cells from OT-I mice and were subsequently immunized with VLV, UV-inactivated VLV, VSV, UV-inactivated VSV, or PBS. One week after immunization, spleen cells from immunized mice were analyzed by flow cytometry and ELISPOT for the presence of SIINFEKL-specific CD8^+^ T cell responses. **a** ELISPOT quantification of SIINFEKL-specific IFN-γ-producing cells. **b** Percentage of IFN-γ-producing CD8^+^ T cells after stimulation with SIINFEKL determined by intracellular cytokine staining. **c** ELISPOT analysis of IFN-γ-producing cells after stimulation with VSV-N peptide. Responses to N by non-inactivated VSV were at the assay upper limit of detection. **d** Frequency of IFN-γ-producing CD8^+^ T cells after stimulation with VSV-N peptide. Data are expressed as mean ± standard deviation. *p < 0.05, **p < 0.01, ****p < 0.0001 compared to PBS control by one-way ANOVA test, *n* = 6.

Since VLV activates DCs in vitro in the absence of viral replication, we next asked whether UV-inactivated VLV could activate antigen-specific immune responses in vivo. We transferred OT-I splenocytes to naïve mice to increase the pool of OVA-specific CD8^+^ T cells, and then 4 h later infected mice with either VLV, VSV, PBS, UV-inactivated VLV, or UV-inactivated VSV. One week after immunization, we quantified OVA-specific CD8^+^ T cell responses. Both VLV and VSV generated OVA-specific CD8^+^ T cells (Fig. 7a, b), and consistent with observations in vitro, VSV more efficiently activated CD8^+^ T cell responses. Interestingly, UV-inactivated VLV primed OVA-specific CD8^+^ T cell responses that although lower in magnitude compared to responses generated by replication-competent VLV were still detectable by ELISPOT and flow cytometry (Fig. 7a, b). However, only replication-competent VSV was able to prime CD8^+^ T cells, and this property was intrinsic to the virus and was not dependent on the antigen, as similar results were observed with VSV-N protein-specific responses (Fig. 7c, d). These results highlight the differences in immune priming with VLV and VSV in vivo.

### cDC1 are necessary for UV-inactivated VLV-induced CD8^+^ T cell responses

**Fig. 8:**
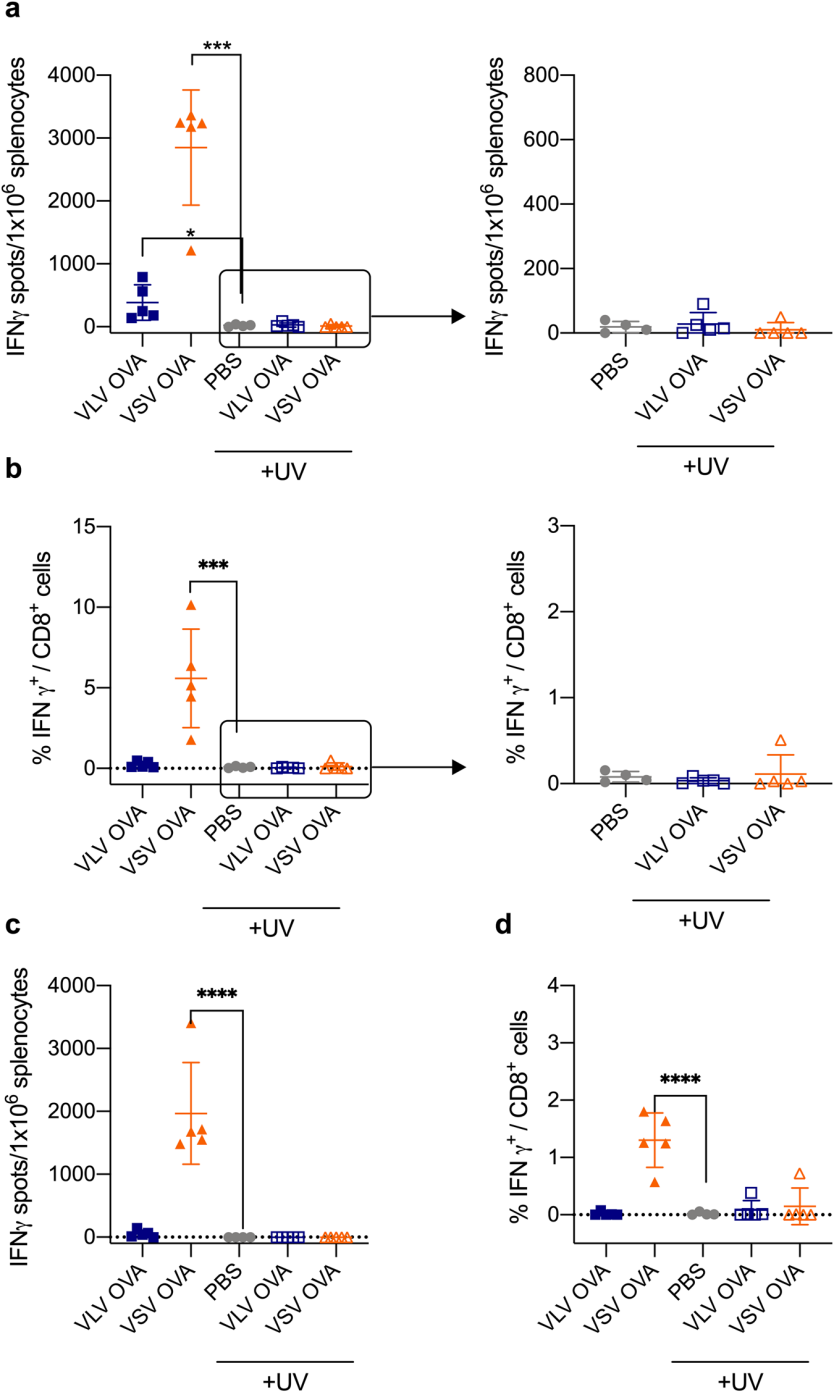
The cDC1 subset is necessary for UV-inactivated VLV-induced CD8^+^ T cell responses. Batf3^**−/−**^ mice received CD8^+^ T cells from OT-I mice and were subsequently immunized with VLV, UV-inactivated VLV, VSV, UV-inactivated VSV, or PBS. One week later, splenocytes were collected and stimulated with the SIINFEKL epitope. **a** ELISPOT quantification of SIINFEKL-specific IFN-γ producing cells. **b** Percentage of IFN-γ-producing CD8^+^ T cells after stimulation with SIINFEKL determined by intracellular cytokine staining. **c** ELISPOT analysis of IFN-γ-producing cells after stimulation with VSV-N peptide. **d** Frequency of IFN-γ-producing CD8^+^ T cells after stimulation with VSV-N peptide. Data are expressed as mean ± standard deviation. *p < 0.05, ***p < 0.001 compared to PBS control using a one-way ANOVA test, *n* = 4–5.

**Fig. 9:**
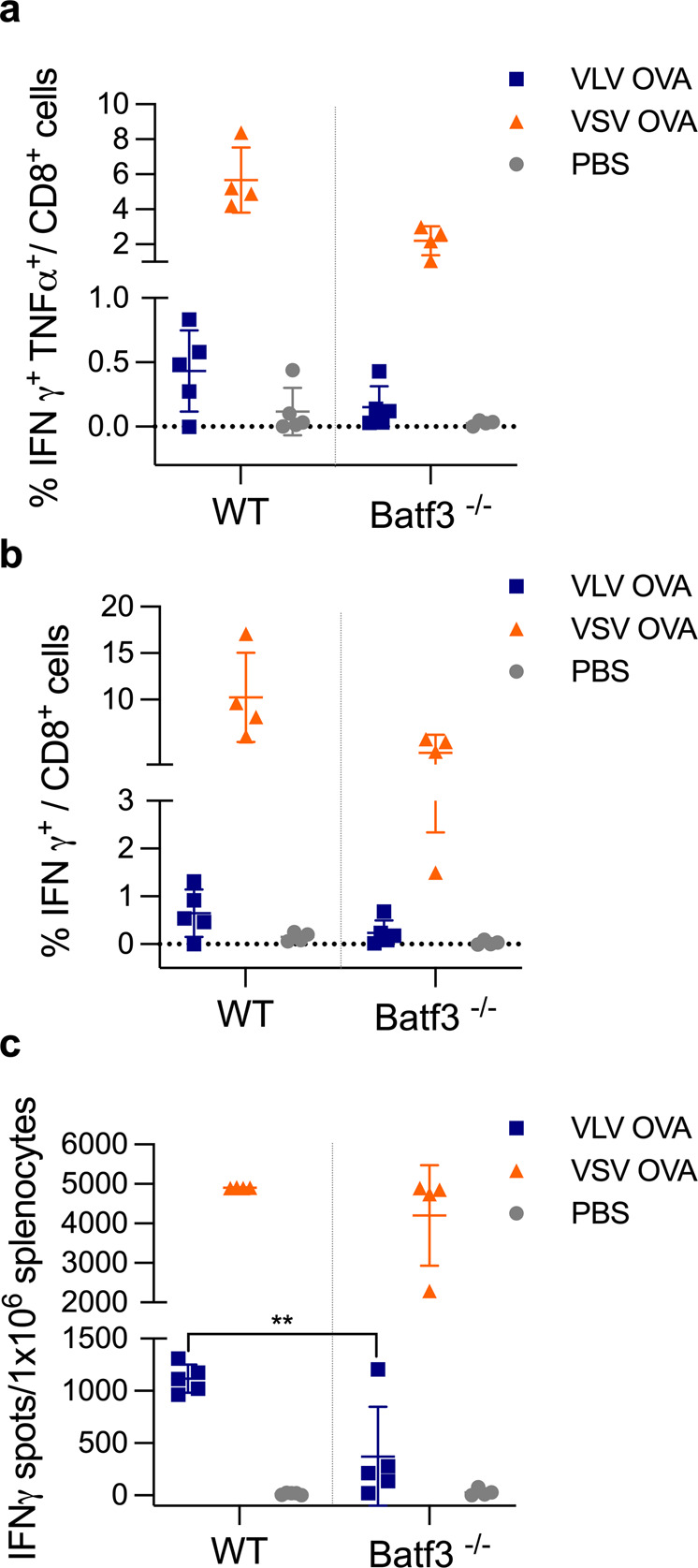
The cDC1 subset contributes to VLV-induced CD8^+^ T cell responses. WT and Batf3^−/−^ mice received CD8^+^ T cells from OT-I mice and were subsequently immunized with VLV, VSV, or PBS. One week later, splenocytes were collected and stimulated with SIINFEKL peptide. **a** Intracellular cytokine staining of IFN-γ- and TNF-α-producing CD8^+^ T cells after stimulation with SIINFEKL. **b** Frequency of IFN-γ-producing CD8^+^ T cells after stimulation with SIINFEKL. **c** ELISPOT quantification of SIINFEKL-specific IFN-γ producing cells. Responses to VSV OVA were near the assay upper limit of detection. Data are expressed as mean ± standard deviation. **p < 0.01 compared to WT mice by two-way ANOVA, *n* = 4–5.

Replication-deficient VLV can activate DCs in vitro and generate antigen-specific CD8^+^ T cells in vivo. The higher infection levels, stronger activation, and lower cell death of Flt3L DCs compared to GM-CSF APCs after viral infection led us to focus on DCs as the most likely APC involved in T cell priming. Because cDC1 is the most relevant antigen-presenting cell type for priming CD8^+^ T cells, either through direct presentation or cross-presentation, we investigated the role of cDC1 in stimulating VLV-induced CD8^+^ T cells using Batf3^−/−^ mice that lack the cDC1 subset^55^. To increase the pool of OVA-specific CD8^+^ T cells, we transferred OT-I CD8^+^ T cells to naïve Batf3^−/−^ mice, and then infected the mice with either VLV, VSV, PBS, UV-inactivated VLV, or UV-inactivated VSV. One week after immunization, we measured the presence of OVA-specific CD8^+^ T cells. In the absence of cDC1, UV-inactivated VLV failed to activate OVA-specific CD8^+^ T cells (Fig. 8, Supplementary Fig. 5), indicating that replication-deficient VLV generates CD8^+^ T cell responses through cDC1. To determine the relative importance of cDC1 in VLV-induced responses, we vaccinated Batf3^−/−^ and WT mice with VLV, VSV, or PBS. Mice that lacked cDC1 had an impaired ability to activate antigen-specific responses after VLV infection (Fig. 9); CD8^+^ T cell responses were decreased in magnitude, and although not statistically significant, there was also a reduction in polyfunctional T cells (Fig. 9a). In contrast, the absence of cDC1 in VSV-induced responses appeared to mostly affect the production of CD8^+^ T cells expressing both TNF-α and IFN-γ (Fig. 9a).

### UV-inactivated VLV induces functional antigen-specific CD8^+^ T cells

**Fig. 10:**
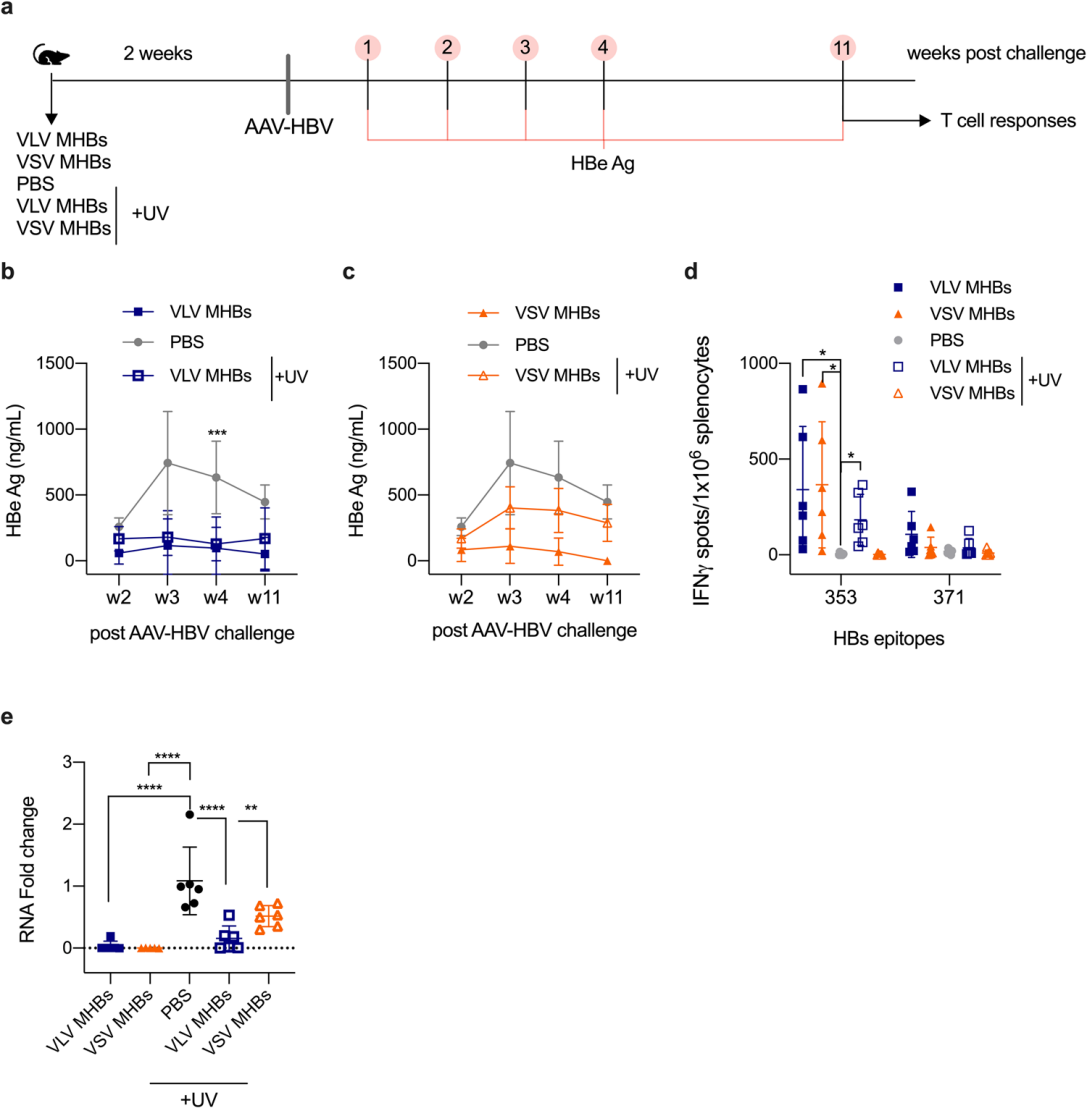
UV-inactivated VLV induces antigen-specific CD8^+^ T cells that are functional. **a** C57BL/6 mice were immunized with VLV or UV-inactivated VLV expressing MHBs antigen, VSV or UV-inactivated VSV expressing MHBs antigen, or PBS. Two weeks after immunization mice were challenged with AAV-HBV (3 × 10^10^ genome copies per mouse). Serum HBe antigen levels were measured in mice that were immunized with **b** VLV or UV-inactivated VLV, or immunized with **c** VSV or UV-inactivated VSV. **d** ELISPOT quantification of MHBs-specific IFN-γ producing cells in the spleen of vaccinated mice at week 11 after AAV-HBV challenge. **e** HBV liver RNA levels were measured by RT-qPCR and normalized to GAPDH 11 weeks after AAV-HBV challenge. Data are expressed as mean ± standard deviation. *p < 0.05, ***p < 0.001 compared to PBS control, by multiple *t*-test or one-way ANOVA test, *n* = 6.

Our data suggest that VLV can activate CD8^+^ T cell responses in the absence of viral replication. Further investigating the functionality of UV-inactivated VLV-induced responses required a model dependent upon CD8^+^ T cell responses for immune control. In previous studies, we found that VLV and VSV expressing the HBV MHBs surface glycoprotein activate CD8^+^ T cell responses that protect mice from the establishment of HBV replication^37,45–48^. Therefore, we vaccinated WT mice, and two weeks later, challenged them with AAV-HBV and measured HBV antigen expression over time (Fig. 10a). Mice that received VLV or UV-inactivated VLV were protected from AAV-HBV challenge, as measured by reduction of an HBV replication marker, HBeAg (Fig. 10b). However, only mice that received VSV but not UV-VSV were protected from HBV challenge (Fig. 10c). Furthermore, we evaluated the presence of MHBs-specific CD8^+^ T cell responses 11 weeks after AAV-HBV challenge and found HBV-specific responses in those mice that received VLV, VSV, and UV-inactivated VLV, but not UV-inactivated VSV (Fig. 10d). Consistent with these results, HBV liver RNA was also significantly decreased in mice that were vaccinated with VLV, UV-VLV, and VSV (Fig. 10e). These results indicate that UV-inactivated VLV induces functional CD8^+^ T cell responses.

## Discussion

The extent to which viral vectors interact with DCs can influence the outcome of the immune response. The mechanism of immune priming may also impact how a vaccine platform is clinically deployed. If the goal is to generate protective immunity mediated by antibodies, it is necessary for a vaccine to elicit strong B cell responses. If CD8^+^ T cells are the end goal, it is important to understand how the vaccine will generate this response. For example, cross-presentation mechanisms are affected in aged populations^56^, so a vaccine that requires this pathway might not be effective in the elderly. Also, it is necessary to recognize whether a platform might be better used for a specific pathogen, or to elicit better responses to antigens with different degrees of protein stability^57^. Some platforms might favor CD8^+^ T cell responses over antibodies, which can be understood by the way that they interact with immune cells and affect CD4^+^ T cell differentiation into Th1, Th2, or Th17 cells. The nature of the viral vector can also impact the interaction with DCs^17^. It is known that different DCs play key roles in activating different cellular responses^58^, and so the interaction of viral vectors with these cells has the capacity to influence the quality of the response, as well as long-term memory.

Our results show differences in DC activation with viral vectors that utilize the same entry receptor (Fig. 1), highlighting that viral tropism is not the only important factor for DC-virus interaction and activation. The lack of cell activation with VSV could imply that the virus is causing cell death; however, there were no differences between the cell death induced by VLV and VSV in Flt3L DCs (Fig. 2). The limited cell activation observed with VSV was previously reported^54^, and is due to VSV modulating the cellular response through the M protein^24^, which helps VSV avoid innate immunity by both interrupting cellular transcription and blocking mRNA export from the nucleus^24^. VSV-M binds to the nucleoporin Nup98 to block mRNA export through the nuclear pore^59–61^. VLV encodes the SFV nonstructural proteins, of which the nsP2 protein was reported to inhibit IFN production^62^. However, our previous results showed significant IFN induction with this platform^63^, which would suggest a possible mutation in nsP2 during the evolution of the VLV platform in IFN-deficient BHK-21 cells that abrogates this IFN antagonism function. Furthermore, our data showed that IFN is not critical for DC activation following VLV infection (Fig. 5) and therefore, even if nsP2 were to interfere with IFN signaling, it would not affect DC activation. As the only VLV structural protein is VSV-G, it does not encode a protein with a function like VSV-M and is essentially a replicating naked RNA, which can be detected by the immune system to quickly activate the immune response to a greater extent in the absence of viral immunomodulators. Another possibility is that VLV could be generally less efficient than VSV at infecting cells since the vesicles are produced by an unnatural mechanism, so that at an equivalent MOI, there is an excess of noninfectious particles that could lead to differences in cell activation. Although we did not investigate the subsets of cells infected by the vectors, it is likely that the differences in infection and activation between Flt3L and GM-CSF cells are due to the small number of DCs present in GM-CSF cultures compared to those in Flt3L^49,51,64^.

The ability of viruses to induce bystander activation was previously observed for other viral vectors^65^, and it is thought to be an important quality for a vaccine vector. Our data showed that VLV infection causes bystander activation of DCs, while VSV does not (Fig. 4). Interestingly, this was independent of IFN signaling (Fig. 5), contrary to a previous finding with vaccinia virus^65^. It is possible that this bystander activation is due to other soluble mediators, such as TNF-α, or it could be due to a cell–cell interaction. Since VSV did not promote bystander activation, it is possible that this virus actively blocks this process. The differences could be additionally explained by the unique replication of the vectors interacting with immune sensors.

Considering all the differences between the two platforms, it was essential to address the role of viral replication on DC activation. When considering viral vectors as vaccines, the ability to replicate is typically thought to confer an immunogenic advantage since replication may lead to prolonged antigen expression and the generation of PAMPs. However, replication can also be considered a safety concern, so replication-deficient platforms are often preferred. We found that VLV infection activates DCs in the absence of replication (Fig. 6). The ability of VLV to activate DCs depends on VSV-G (Fig. 4c), which suggests activation cannot be achieved simply by endocytosis. It is likely that when the vector enters the cell, the exposed viral RNA is detected by PRRs in the cytoplasm or by endosomal TLR7. In this case, it is possible that activation with UV-inactivated VLV proceeds through a different pathway than replication-competent VLV; inactivated replication-deficient VLV won’t be able to form the replication spherules characteristic of VLV replication, and therefore will likely interact with other immune receptors in the cytoplasm.

In vitro proliferation of antigen-specific CD8^+^ T cells co-cultured with infected DCs demonstrated a higher capacity for CD8^+^ T cell stimulation with VSV-infected DCs (Fig. 3). Consistent with these data, in vivo priming with VSV rendered stronger OVA-specific responses than VLV (Fig. 7). Interestingly, both MHC I and B2M were induced to higher levels in VLV-infected DCs. Although this result might appear contradictory at first, the increase observed in MHC II and CD86 might play a more important role in activating CD4^+^ T cell responses compared to CD8^+^ T cell responses. We did not study the capacity of the vectors to prime CD4^+^ T cell responses, but it would be interesting to determine whether the differences in CD8^+^ T cell priming are due to CD4^+^ helper T cell skewing from differential functional activation of DCs after infection. For example, VLV might promote the production of cytokines that contribute to Th2 differentiation, and therefore be a weaker inducer of CD8^+^ T cell responses. Additionally, our results suggest that VSV induction of CD8^+^ T cell responses does not require cDC1 for CD8^+^ T cell priming (Figs. 8 and 9), which would indicate a role for other antigen-presenting cells, such as other DC subsets, or more likely macrophages, as these cells have been shown to be important for VSV control^66^. A caveat from our in vitro study (Fig. 3a–d) was the use of total splenocytes and not purified CD8^+^ T cells; therefore, other cell types may have acted as antigen-presenting cells. Nevertheless, our results suggest that DCs are the main antigen-presenting cells for VLV, but not VSV, since even though lower DC activation was achieved after VSV infection, VSV led to the strongest CD8^+^ T cell responses (Figs. 3 and 7–10). This is consistent with the role of other immune cells, such as macrophages, in CD8^+^ T cell priming with VSV. Furthermore, even though VLV induced greater DC activation and there was also a significant bystander effect on activation, it is known that not all activated cells prime T cell responses equally^67,68^. Further studies to investigate the nature of antigen presentation after VLV infection will aim to better understand this process.

UV-inactivated VLV primed antigen-specific CD8^+^ T cells (Fig. 7), even though those responses were not as great in magnitude compared to replication-competent VLV. Interestingly, VSV required replication to both activate DCs and prime CD8^+^ T cell responses, which indicates that the vectors activate CD8^+^ T cell responses through different pathways. The role of cDC1 in UV-VLV activation of CD8^+^ T cells (Figs. 8 and 9) could mean that cDC1 uptake and cross-present antigen from other cells, but direct infection of these cells cannot be ruled out. When originally described, VLV was found not to carry any protein but VSV-G; however, our results from UV-inactivation could indicate the presence of OVA protein in those vesicles^35^. It is also possible that even though the vectors are UV-inactivated, the RNA is partially transcribed/translated but incomplete, and immunogenic products are synthesized, and CD8^+^ T cell priming still occurs. Future studies to elucidate the specific way in which UV-inactivated VLV prime CD8^+^ T cells will be needed.

The ability of UV-inactivated VLV to generate CD8^+^ T cell responses was unexpected, but it was possible that the quality of the responses was not adequate and would not be functional. Strikingly, when we challenged vaccinated mice with AAV-HBV, mice that received either VLV or UV-VLV were protected from the challenge, but only those that received VSV but not UV-VSV were protected (Fig. 10). This result indicates that despite being low in magnitude, replication-deficient VLV generates functional CD8^+^ T cell responses. Future studies will be needed to determine if these effector cells can develop into memory and protect from further encounters with a pathogen.

In summary, in this study, we showed that RNA viral vectors with identical entry pathways but distinct replication mechanisms differentially infect and activate DCs, leading to unique CD8^+^ T cell priming. The mechanism of viral vector replication has a key impact on the function and priming of CD8^+^ T cells, and further understanding this effect will aid in the design of better viral vector-based vaccines.

## Methods

### BMDC generation

Six- to eight-week-old female mice were euthanized, and bone marrow cells were obtained by flushing cells from both femurs and tibias. Cells were collected in RPMI 1640 medium supplemented with 10% FBS, 100 U/mL Penicillin and 100 μg/mL streptomycin, 100 nM non-essential amino acids, 1 mM sodium pyruvate, and 55 nM β-mercaptoethanol. Flt3L-derived DCs give rise to a heterogeneous population of both cDCs and pDCs^50,51^. BMDC differentiation with GM-CSF generates a mixture of APCs that includes macrophages and DCs with an inflammatory monocyte-derived phenotype^49,64,69^. For Flt3L BMDCs, cells were seeded in 6 well plates at a concentration of 2 × 10^6^ cells/mL in the presence of 100 ng Flt3L/mL (Peprotech)^52^. GM-CSF BMDCs were generated by plating 1 × 10^6^ cells/mL in 10 cm dishes with 10 ng GM-CSF/mL (Peprotech)^49,52^. Cells were incubated at 37 °C for 8 days, and media containing Flt3L or GM-CSF was supplemented at day 4. On day 8, cells were collected for infection.

### Viruses

The VSV GFP, VSV OVA, VSV MHBs, VLV MHBs, and VLV GFP vectors were previously reported^38,45,63,70,71^. VLV-OVA was constructed by cloning a codon-optimized open reading frame encoding Ovalbumin between the *Pac*I and *Sbf*I sites of pCMV-SFVT2AG^37^. VSV and VLV stocks were generated and propagated using BHK-21 cells, concentrated, and titers were determined by infection of BHK-21 cells with serial dilutions of the virus stocks in a plaque-forming assay using standard protocols.

### In vitro infection of BMDCs

Cells were collected, pelleted by centrifugation, and resuspended in 5 mL of serum-free RPMI. After cell numbers were determined, cells were infected with several multiplicities of infection (MOI) for each viral vector. Viral vectors encoding GFP were utilized to measure infection by flow cytometry. The initial infection was carried out at 37 °C in serum-free media, then cells were supplemented with complete RPMI media, and the infection was continued for 18 h unless otherwise indicated. To block VSV-G-mediated entry, VLVs were pre-incubated with rabbit polyclonal anti-VSV antibody for 1 h before the infection.

### Intracellular cytokine staining and flow cytometry

Antigen-specific IFN-γ-producing or TNF-α/IFN-γ-producing CD8^+^ T cells were detected by flow cytometry following stimulation with OVA SIINFEKL peptide or VSV nucleocapsid peptide as indicated for each experiment. Spleen cells were harvested, red blood cells were removed by lysis with ACK buffer, and cells were stimulated with peptide for 5 h in the presence of brefeldin and monensin. Cells were stained for surface markers CD8 (1:200; EBioscience #17008183) and CD3 (1:100; BD Biosciences #557984), and intracellular cytokine staining for IFN-γ (1:200; BD Biosciences #554412) and TNF-α (1:100; EBioscience #48732182) was performed using a Fixation/Permeabilization Kit (BD Cytofix/Cytoperm™). DC activation was determined by flow cytometry analysis of MHC II (1:400; BD Biosciences #557000), CD80 (1:200; BD Biosciences #562611), CD86 (1:200; BD Biosciences #560582), and CD11c (1:100; BD Biosciences #550261). Samples were analyzed using a BD LSR II cytometer and FlowJo software.

### Cell death analysis

Apoptosis was assessed by staining the infected cells with annexin V-Pacific blue (Biolegend) for 10 min. The cells were then stained with propidium iodide (PI) for 5 min before analysis by flow cytometry.

### RNA detection by qPCR

Cells were collected, RNA was purified using an RNeasy kit (Qiagen), and cDNA was prepared from equal amounts of RNA using a High Capacity cDNA Reverse Transcription kit (Applied Biosystems). Quantitative PCR was performed using Taqman Fast Advanced Master Mix (Applied Biosystems). Reactions were done using a StepOnePlus real-time PCR system (Applied Biosystems) with StepOne software v2.3. Taqman Assay Mix containing probe and specific primers for mouse GAPDH (Mm99999915_g1), H-2D1/H-2K1 (Mm04208017_mH), and β2-microglobulin (B2M; Mm00437762_m1) (Thermofisher). Gene expression was quantified by the comparative ΔΔC_T_ method. For the RT^2^ profiler PCR assay, RNA was purified utilizing an RNeasy Plus kit, and samples were analyzed using an RT^2^ First-Strand Kit, SYBR Green qPCR master mix, and Dendritic and Antigen Presenting Cell PCR array (Qiagen). For HBV RNA detection, the following sequences were used: HBV probe, 5'-CCT CTT CAT CCT GCT GCT ATG CCT CAT C-3'; antisense, 5'-GAC AAA CGG GCA ACA TAC CTT-3'; sense, 5'-GTG TCT GCG GCG TTT TAT CA-3'^72^.

### Immunizations

All immunizations were done intramuscularly in 50 μL PBS. For immunizations with VSV, mice received 1 × 10^6^ PFU/mouse unless indicated; for VLV, 1 × 10^7^ PFU/mouse was administered. In some experiments, viruses were inactivated using two UV doses of 200 mJ/cm^2^ and 150 mJ/cm^2^. OT-I splenocytes or CD8^+^ T cells were transferred to recipient mice by injecting 200 µL of either 8 × 10^6^ total spleen cells or 5 × 10^5^ splenic CD8^+^ T cells via intravenous injection.

### CD8^+^ T cell purification

Splenic OT-I CD8^+^ T cells were purified utilizing a CD8α^+^ T Cell Isolation Kit (Miltenyi) for untouched separation of cells following the manufacturer’s protocol.

### Mice

C57BL/6 (stock #000664), B6.129S(C)-*Batf3*^*tm1Kmm*^/J (stock #013755; Batf3^−/−^)^55^, B6.129S2-*Ifnar1*^*tm1Agt*^ (stock #32045; IFNAR^−/−^)^73^, and C57BL/6-Tg(TcraTcrb)1100Mjb/J (stock #003831; OT-I)^74^ mice were obtained from The Jackson Laboratory. 129S6/SvEv-*Stat1*^*tm1Rds*^ (STAT1^−/−^)^75^ mice were purchased from Taconic Biosciences. Six- to eight-week-old female and male mice were used for immunogenicity studies; no sex-specific differences were observed. Mice were housed in the Animal Resource Facility at Albany Medical College, and all experiments were done following protocols approved by the Albany Medical College Institutional Animal Care and Use Committee.

### CFSE assays

BMDCs were infected with VLV or VSV expressing OVA. Eighteen hours after infection, cells were washed thoroughly and co-cultured with CFSE-labeled OT-I splenocytes (CellTrace™ CFSE Cell Proliferation Kit, Invitrogen) for 72 h. Proliferation was then assessed by flow cytometry of CD8^+^ cells.

### ELISA

Serum HBeAg was measured by ELISA (International Immunodiagnostics) following the manufacturer’s protocol. Recombinant HBeAg standard was purchased from Fitzgerald Industries. IL-10 expression was measured utilizing a Bio-Rad Bio-Plex Pro mouse cytokine assay.

### ELISPOT assay

IFN-γ-producing CD8^+^ T cells were measured by IFN-γ ELISPOT assay (BD Biosciences)^76^. Briefly, 96 well Millipore Immunospot M200 plates were coated overnight with purified anti-mouse IFN-γ antibody (1:200). Purified splenocytes were resuspended in complete RPMI and seeded for overnight stimulation with peptide epitopes (described below) at 37 °C. After stimulation, plates were washed with water once and PBS-Tween twice followed by a 2 h incubation with biotinylated anti-mouse IFN-γ antibody (1:250) at room temperature. After washing, streptavidin-horseradish peroxidase (HRP) (1:100) was added to wells and incubated for 1 h at room temperature. Following the final washes, 3-amino-9-ethyl-carbazole (AEC) chromogen substrate (BD Biosciences) was added to the wells and developed at room temperature for 30 min to 1 h. Plates were rinsed with water and dried before proceeding with spot quantification. Spot formation was quantified using an automated spot counter (Immunospot, Cellular Technology Ltd).

### Peptide epitopes

T cell stimulation was performed using known CD8^+^ T cell epitopes for OVA, VSV nucleocapsid (N), and HBsAg. SIINFEKL is an immunodominant H-2K^b^ -restricted CD8^+^ T cell epitope from OVA^77^. VSV N was utilized as a control for immune responses to VSV, which comprises amino acids (a.a.) 52-59 (RGYVYQGL) of the N protein and is also specific for H-2K^b^^78^. HBV S 353 is an immunodominant H-2K^b^-restricted HBsAg CD8^+^ T cell epitope consisting of a.a. 353–360 (VWLSVIWM)^79^. HBV S 371 is an immunodominant H-2K^b^-restricted HBsAg CD8^+^ T cell epitope that consists of a.a. 371–378 (ILSPFLPL)^79,80^.

### AAV-HBV transduction

Serotype 8 of adeno-associated virus (AAV) encoding a 1.2-mer HBV genome was prepared by SignaGen. HBV replication was initiated in male C57BL/6 mice with transduction via intravenous injection of 3 × 10^10^ genome copies of AAV-HBV.

### Statistical analysis

Data were analyzed with GraphPad Prism 7 or 8 software using unpaired or multiple two-tail *t*-tests comparing to PBS or uninfected controls. ANOVA tests with Tukey’s multiple comparison tests were also done to assess differences among more than two groups (one-way) or among more than two groups and two variables (two-way). Data points represent distinct samples. For in vitro experiments, we typically performed technical replicates, and experiments were repeated. Means and standard deviations are depicted for each experiment. *P*-values < 0.05 are shown for each experiment.

### Reporting summary

Further information on research design is available in the Nature Research Reporting Summary linked to this article.

## Supplementary information

Supplementary Information

Reporting Summary

## Data Availability

The data that support the findings of this study are available from the corresponding author on reasonable request.
