# Supplementary material for: Virus-based vaccine vectors with distinct replication mechanisms differentially infect and activate dendritic cells
Source: NPJ Vaccines. 2021 Nov 22;6:138. doi: 10.1038/s41541-021-00400-w (PMC8608815; doi:10.1038/s41541-021-00400-w)
Supplement: Supplementary file 1 — Supplementary Information [file 41541_2021_400_MOESM1_ESM.pdf]

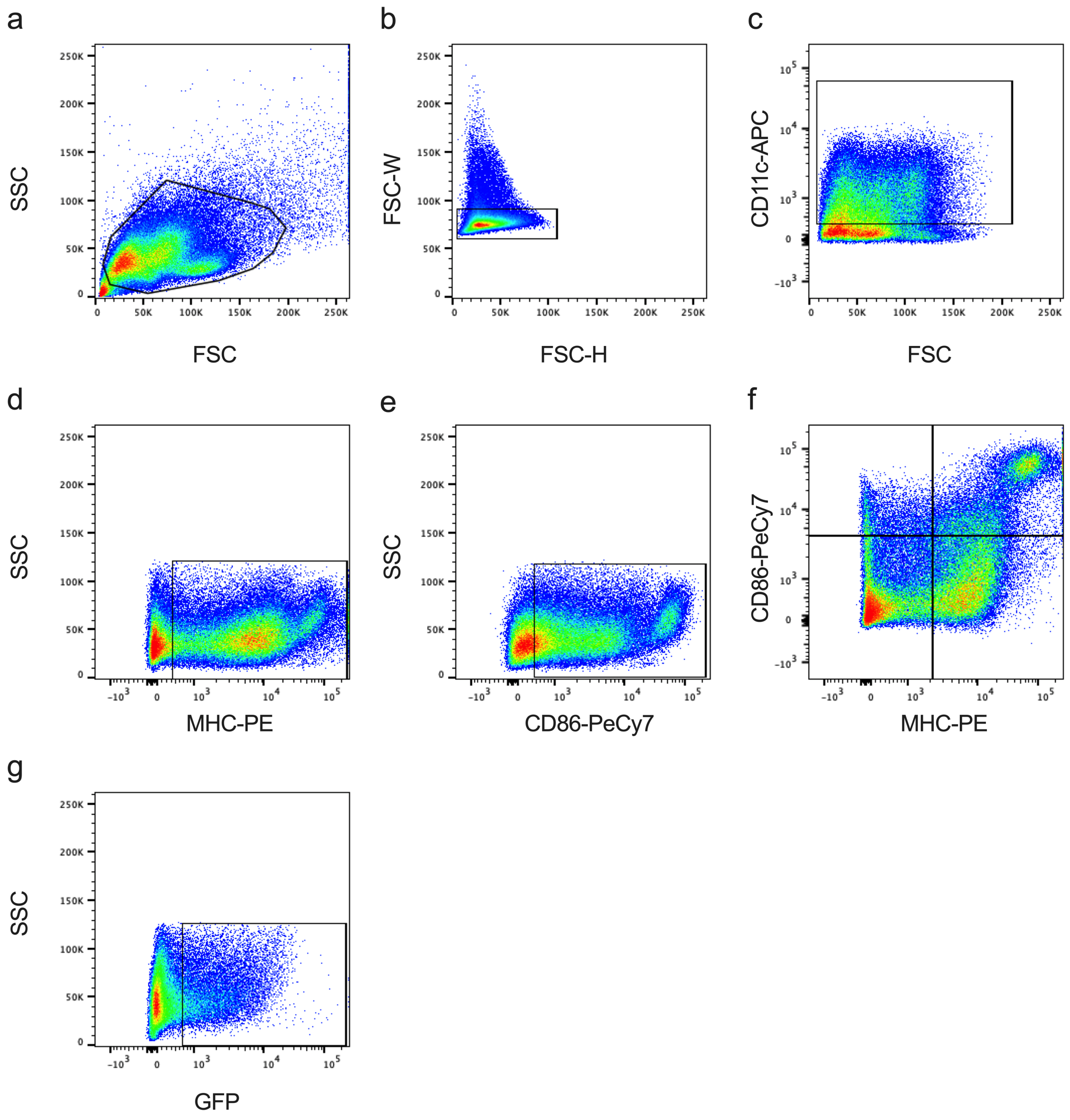


**Supplementary Figure 1. Gating strategy for BMDC in vitro activation.** After BMDCs were infected, activation was measured by flow cytometry. The following gates were used: (a) total cells, (b) singlets, then (c) $CD11c^{+}$ cells were selected. Then, expression of (d) MHC II, (e) CD86, and (f) MHC II and CD86 was determined. (g) Gating of GFP+ cells in some experiments. In some experiments, measurement of CD80 was similarly determined.

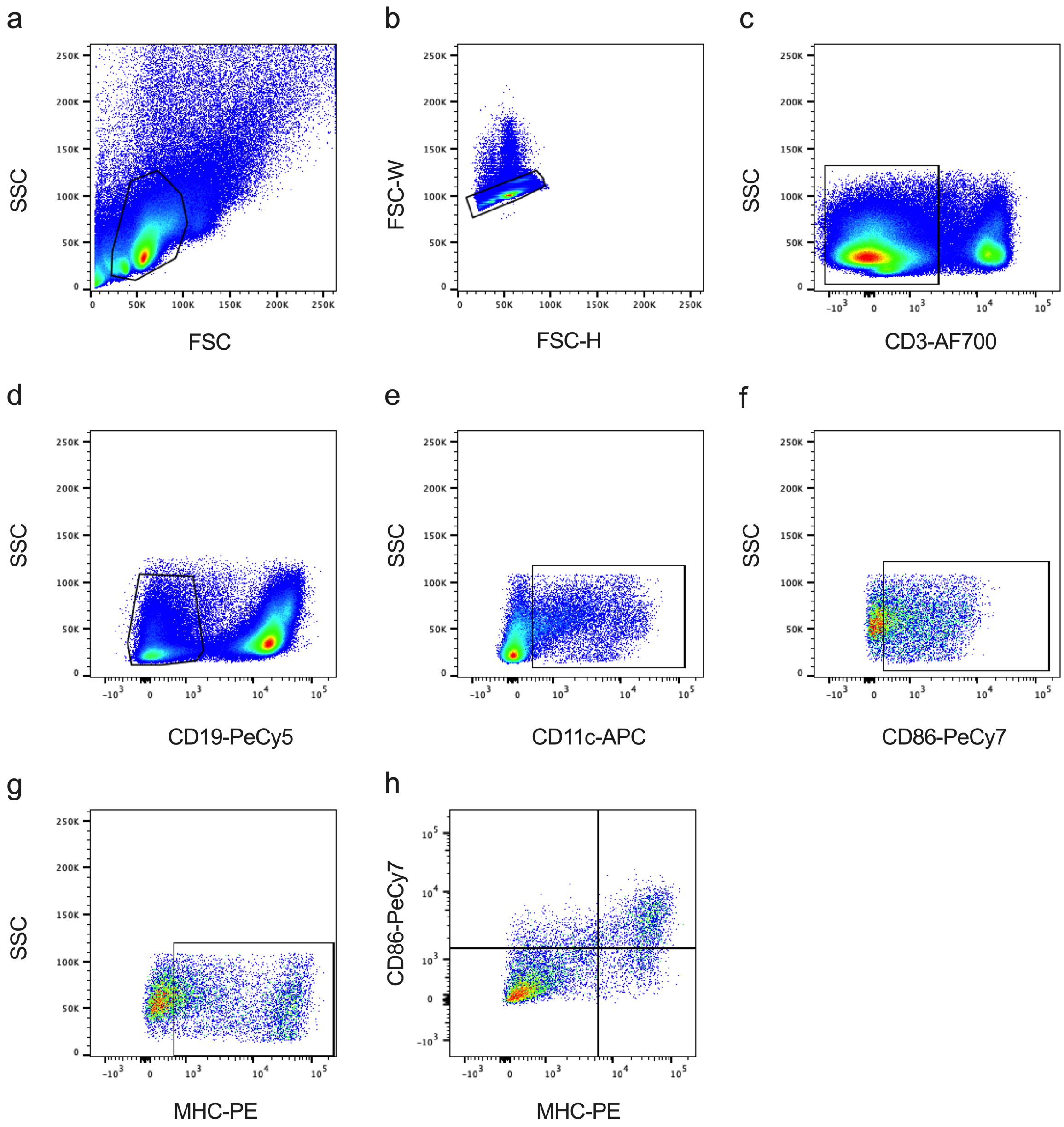


**Supplementary Figure 2. Gating strategy for in vivo activation of APCs.** Splenocytes from infected mice were analyzed by flow cytometry. The following gates were used: (a) total cells; (b) singlets. Then, cells were selected as (c) CD3$^{-}$, (d) CD19$^{-}$, and (e) CD11c$^{+}$. This was followed by analysis of (f) CD86, (g) MHC II, and (h) MHCII$^{+}$CD86$^{+}$ cells.

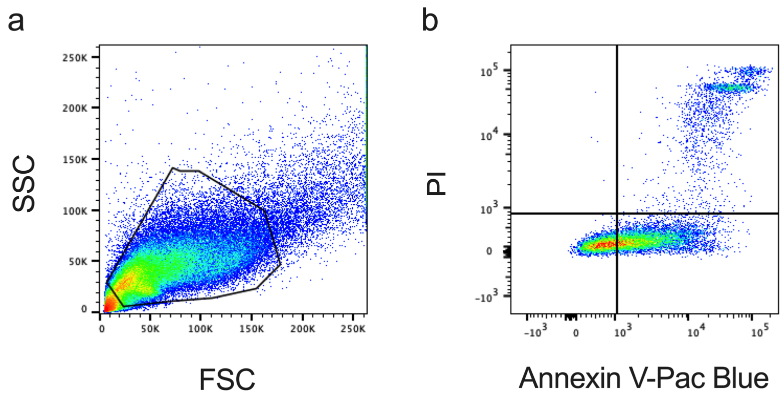


**Supplementary Figure 3. Gating strategy for annexin V and PI staining.** (a) Total cells gate; (b) PI and annexin V.

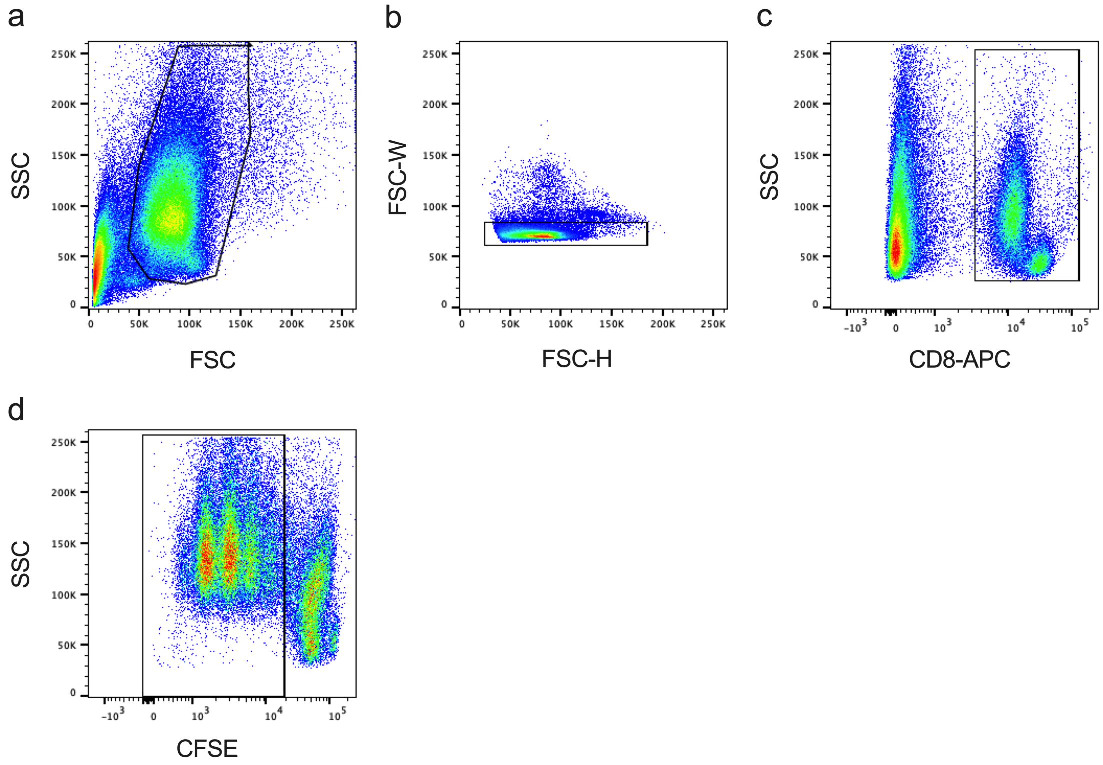


**Supplementary Figure 4. Gating strategy for CFSE staining.** (a) Total cells gate; (b) singlets; (c) CD8+ cells. (d) Total proliferation (CFSE low) in co-cultures of OT-I splenocytes with BMDCs.

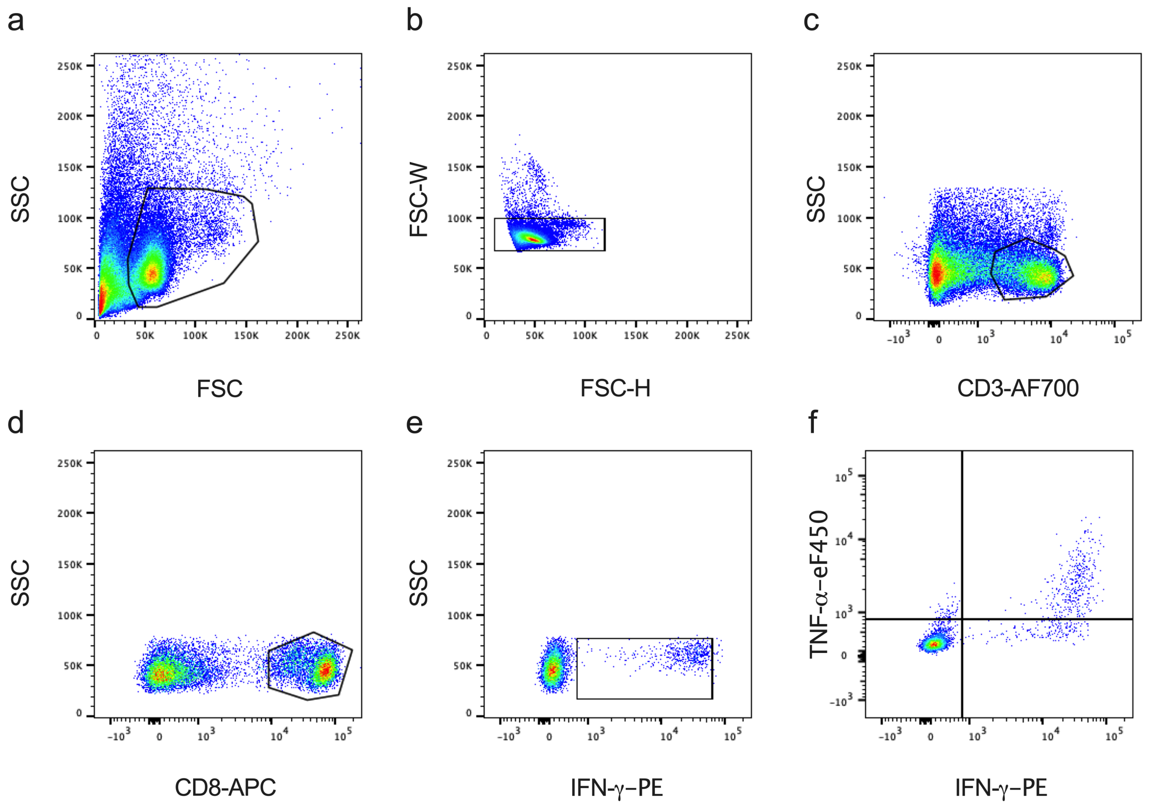


**Supplementary Figure 5. Gating strategy for intracellular cytokine staining.** (a) Total cells gate; (b) singlets; (c) $CD3^+$ cells; (d) $CD8^+$ cells. Then, (e) IFN-γ production and (f) IFN-γ and TNF-α expression were measured.
