# Supplementary material for: Virus-based vaccine vectors with distinct replication mechanisms differentially infect and activate dendritic cells
Source: NPJ Vaccines. 2021 Nov 22;6:138. doi: 10.1038/s41541-021-00400-w (PMC8608815; doi:10.1038/s41541-021-00400-w)
Supplement: Supplementary file 2 — Reporting Summary [file 41541_2021_400_MOESM2_ESM.pdf]

nature research

Corresponding author(s): Michael D. Robek

Last updated by author(s): Sep 26, 2021

Reporting Summary

Nature Research wishes to improve the reproducibility of the work that we publish. This form provides structure for consistency and transparency in reporting. For further information on Nature Research policies, see our Editorial Policies and the Editorial Policy Checklist.

Statistics

For all statistical analyses, confirm that the following items are present in the figure legend, table legend, main text, or Methods section.

n/a	Confirmed	
☐	☒	The exact sample size (*n*) for each experimental group/condition, given as a discrete number and unit of measurement
☐	☒	A statement on whether measurements were taken from distinct samples or whether the same sample was measured repeatedly
☐	☒	The statistical test(s) used AND whether they are one- or two-sided<br>*Only common tests should be described solely by name; describe more complex techniques in the Methods section.*
☒	☐	A description of all covariates tested
☐	☒	A description of any assumptions or corrections, such as tests of normality and adjustment for multiple comparisons
☐	☒	A full description of the statistical parameters including central tendency (e.g. means) or other basic estimates (e.g. regression coefficient) AND variation (e.g. standard deviation) or associated estimates of uncertainty (e.g. confidence intervals)
☐	☒	For null hypothesis testing, the test statistic (e.g. *F*, *t*, *r*) with confidence intervals, effect sizes, degrees of freedom and *P* value noted<br>*Give P values as exact values whenever suitable.*
☒	☐	For Bayesian analysis, information on the choice of priors and Markov chain Monte Carlo settings
☒	☐	For hierarchical and complex designs, identification of the appropriate level for tests and full reporting of outcomes
☒	☐	Estimates of effect sizes (e.g. Cohen's *d*, Pearson's *r*), indicating how they were calculated

Our web collection on statistics for biologists contains articles on many of the points above.

Software and code

Policy information about availability of computer code

Data collection	ImmunoSpot Software v7.0 (Cellular Technology Limited)<br>FACSDiva v8.0 (BD)<br>StepOnePlus Software v2.3 (Applied Biosystems)<br>SoftMax Pro v7.0 (Molecular Devices)
Data analysis	Prism v7 and 8 (GraphPad)<br>FlowJo v10 (BD)

For manuscripts utilizing custom algorithms or software that are central to the research but not yet described in published literature, software must be made available to editors and reviewers. We strongly encourage code deposition in a community repository (e.g. GitHub). See the Nature Research guidelines for submitting code & software for further information.

Data

Policy information about availability of data

All manuscripts must include a data availability statement. This statement should provide the following information, where applicable:

- Accession codes, unique identifiers, or web links for publicly available datasets
- A list of figures that have associated raw data
- A description of any restrictions on data availability

The data that support the findings of this study are available from the corresponding author on reasonable request.

Field-specific reporting

Please select the one below that is the best fit for your research. If you are not sure, read the appropriate sections before making your selection.

☒ Life sciences ☐ Behavioural & social sciences ☐ Ecological, evolutionary & environmental sciences

For a reference copy of the document with all sections, see nature.com/documents/nr-reporting-summary-flat.pdf

Life sciences study design

All studies must disclose on these points even when the disclosure is negative.

Sample size	For mouse experiments, we typically aimed for a sample size of 6 animals per group, which allows the measurement of 50% differences of a mean response assuming a standard deviation of 30% using a t-test with an alpha error of 0.05 and a statistical power greater than 0.80.
Data exclusions	No data were excluded.
Replication	For in vitro experiments, we typically performed technical replicates and experiments were repeated.
Randomization	Mice were randomly allocated to each experimental group.
Blinding	No blinding was used.

Reporting for specific materials, systems and methods

We require information from authors about some types of materials, experimental systems and methods used in many studies. Here, indicate whether each material, system or method listed is relevant to your study. If you are not sure if a list item applies to your research, read the appropriate section before selecting a response.

Materials & experimental systems

n/a	Involved in the study
☐	☒ Antibodies
☐	☒ Eukaryotic cell lines
☒	☐ Palaeontology and archaeology
☐	☒ Animals and other organisms
☒	☐ Human research participants
☒	☐ Clinical data
☒	☐ Dual use research of concern

Methods

n/a	Involved in the study
☒	☐ ChIP-seq
☐	☒ Flow cytometry
☒	☐ MRI-based neuroimaging

Antibodies

Antibodies used	MHCII (BD, 557000)<br>CD80 (BD, 562611)<br>CD86 (BD, 560582)<br>CD11c (BD, 550261)<br>CD3 (BD, 557984)<br>CD8 (EBioscience, 17008183)<br>CD19 (EBioscience, 15019381)<br>IFNgamma (BD, 554412)<br>TNFalpha (EBioscience, 48732182)
Validation	Validated by the manufacturers.

Eukaryotic cell lines

Policy information about cell lines

Cell line source(s)	BHK-21 cells were obtained from ATCC and the laboratory of Dr. John Rose (Yale University).
Authentication	No authentication was performed.
Mycoplasma contamination	Cells were not tested for Mycoplasma.

Commonly misidentified lines (See ICLAC register)	N/A

Animals and other organisms

Policy information about studies involving animals; ARRIVE guidelines recommended for reporting animal research

Laboratory animals	C57BL/6 (stock #000664), B6.129S(C)-Batf3tm1Kmm/J (stock #013755), B6.129S2-Ifnar1tm1Agt (stock #32045), and C57BL/6-Tg(TcraTcrb)1100Mjb/J (stock #003831) mice were obtained from The Jackson Laboratory. 129S6/SvEv-Stat1tm1Rds mice were purchased from Taconic Biosciences. Six- to eight-week-old female and male mice were used for immunogenicity studies; no sex-specific differences were observed. Male mice were utilized for the HBV experiment due to higher virus production in males.
Wild animals	The study did not involve wild animals.
Field-collected samples	The study did not involve field-collected samples.
Ethics oversight	Mice were maintained in the AAALAC-accredited Animal Resource Facility at Albany Medical College (AMC) and were handled with procedures approved by the AMC Institutional Animal Care and Use Committee.

Note that full information on the approval of the study protocol must also be provided in the manuscript.

Flow Cytometry

Plots

Confirm that:

☒ The axis labels state the marker and fluorochrome used (e.g. CD4-FITC).

☒ The axis scales are clearly visible. Include numbers along axes only for bottom left plot of group (a 'group' is an analysis of identical markers).

☒ All plots are contour plots with outliers or pseudocolor plots.

☒ A numerical value for number of cells or percentage (with statistics) is provided.

Methodology

Sample preparation	For BMDC analysis, cells were collected from culture, centrifuged, washed, and resuspended in staining buffer. For analysis of mouse spleen cells, splenocytes were prepared as described in Reynolds et al. 2017. An ELISPOT-based assay to measure HBV-specific CD8+ T cell responses in immunocompetent mice. In H. Guo and A. Cuconati (ed.), Hepatitis B Virus: Methods and Protocols, 1540:237-247. Springer Press, New York, NY. Cells were prepared from mouse lymph nodes using a similar procedure as the spleen, but with the addition of a collagenase digestion step.
Instrument	BD LSR II cytometer
Software	FACSDiva, FlowJo
Cell population abundance	N/A
Gating strategy	For BMDC experiments, the FSC/SSC gate was drawn to avoid cell debris. An FSC-W vs FSC-H single cell gate was then utilized to eliminate doublets. CD11c+ cells were gated utilizing single stain and unstained controls. For some experiments, GFP+ cells were also gated utilizing GFP alone and no GFP controls. Activation marker gates were drawn in a similar way. Fluorescence minus one controls were initially utilized for gate design. For in vivo DC experiments, the lymphocyte gate was drawn, then CD3 and CD19 negative cells were gated followed by CD11c+ cells, after which CD86 and MHC II markers were analyzed. For T cell analysis, the gating strategy was as follows: lymphocyte gate, single cell gate, CD3+, CD8+, then IFNgamma+ or IFNgamma+TNFalpha+. For cell death anaysis, cells were gated for FSC vs SSC, followed by Annexin V and PI staining. For proliferation analysis, cells were gated for FSC vs SSC, singlets, CD8, and CFSE.

☒ Tick this box to confirm that a figure exemplifying the gating strategy is provided in the Supplementary Information.
